# High Levels of Post-Abortion Complication in a Setting Where Abortion Service Is Not Legalized

**DOI:** 10.1371/journal.pone.0166287

**Published:** 2017-01-06

**Authors:** Tadele Melese, Dereje Habte, Billy M. Tsima, Keitshokile Dintle Mogobe, Kesegofetse Chabaesele, Goabaone Rankgoane, Tshiamo R. Keakabetse, Mabole Masweu, Mosidi Mokotedi, Mpho Motana, Badani Moreri-Ntshabele

**Affiliations:** 1 Department of Obstetrics and Gynaecology, University of Botswana, Gaborone, Botswana; 2 Consultant Public Health Specialist, Addis Ababa, Ethiopia; 3 Department of Family Medicine and Public Health, University of Botswana, Gaborone, Botswana; 4 School of Nursing, University of Botswana, Gaborone, Botswana; 5 Botswana Ministry of Health, Gaborone, Botswana; Stellenbosch University, SOUTH AFRICA

## Abstract

**Background:**

Maternal mortality due to abortion complications stands among the three leading causes of maternal death in Botswana where there is a restrictive abortion law. This study aimed at assessing the patterns and determinants of post-abortion complications.

**Methods:**

A retrospective institution based cross-sectional study was conducted at four hospitals from January to August 2014. Data were extracted from patients’ records with regards to their socio-demographic variables, abortion complications and length of hospital stay. Descriptive statistics and bivariate analysis were employed.

**Result:**

A total of 619 patients’ records were reviewed with a mean (SD) age of 27.12 (5.97) years. The majority of abortions (95.5%) were reported to be spontaneous and 3.9% of the abortions were induced by the patient. Two thirds of the patients were admitted as their first visit to the hospitals and one third were referrals from other health facilities. Two thirds of the patients were admitted as a result of incomplete abortion followed by inevitable abortion (16.8%). Offensive vaginal discharge (17.9%), tender uterus (11.3%), septic shock (3.9%) and pelvic peritonitis (2.4%) were among the physical findings recorded on admission. Clinically detectable anaemia evidenced by pallor was found to be the leading major complication in 193 (31.2%) of the cases followed by hypovolemic and septic shock 65 (10.5%). There were a total of 9 abortion related deaths with a case fatality rate of 1.5%. Self-induced abortion and delayed uterine evacuation of more than six hours were found to have significant association with post-abortion complications (p-values of 0.018 and 0.035 respectively).

**Conclusion:**

Abortion related complications and deaths are high in our setting where abortion is illegal. Mechanisms need to be devised in the health facilities to evacuate the uterus in good time whenever it is indicated and to be equipped to handle the fatal complications. There is an indication for clinical audit on post-abortion care to insure implementation of standard protocol and reduce complications.

## Introduction

Post-abortion care and contraception are some of the oldest sexual and reproductive health care services, dating back to ancient Roman, Grecian and Egyptian history. Different abortion techniques used by Egyptians were documented in the ancient Ebers Papyrus in 1550 BC [1, 2]. Despite this unintended pregnancy due to an unmet need for contraception and unsafe abortion remains one of the leading causes of maternal death in the 21^st^ Century. Countries with restrictive abortion laws carry the major share of this burden. In such situations it has been reported that one in four pregnancies are unintended; some countries have an unmet need as high as 51% leading to unintended pregnancy which is a major risk for unsafe induced abortion. In such countries unsafe induced abortion is under-estimated as only 4% of women of reproductive age reported the incident, because of criminalization and social stigma [3]. The current understanding is that countries in the developing world have a rate of unsafe induced abortion cases as high as 55% versus 3% in the developed nations [4].

Globally an estimated 43.8 million abortions were induced in 2008, of which 49% were unsafe; in Africa almost all (97%) of induced abortion is unsafe. The induced abortion rate in 2008 was 28 per 1000 women aged 15–44 years. Unsafe induced abortion is very likely to lead to multiple complications resulting in maternal disabilities and deaths. Patients with both spontaneous and induced abortion might be admitted for post-abortion care as if they are having spontaneous miscarriages, in situations where abortion is illegal [4–6]. In Botswana it is not only the abortion law which influences limited access to safe abortion care but socio-cultural beliefs are also found to be an important barrier to safe post-abortion care[7].

Evidence has shown that unsafe abortion related maternal deaths have been decreasing between 1990 and 2008 worldwide. However the estimated incidence of unsafe abortion globally increased from 19.7 million to 21.6 million, and in Africa from 5.5 million to 6.2 million for the years 2003 to 2008 [8]. Unsafe abortion accounted for 13% (1 in 8) of maternal deaths globally in 2008–2009. A total of 68,000 women died from complications of unsafe abortion in the same year. In Africa, in the same year, there were 29,000 maternal deaths attributable to abortion related complications with a ratio of 470 deaths per 100,000 abortion cases. Each year, 5 million women who survive unsafe abortion will end up with long term complications. It is impossible to reach the target set for Sustainable Development Goal (SDG) 3 [9] without improving and standardizing abortion care, as well as increasing availability of modern contraceptives to prevent unintended pregnancies which are likely to lead to induced abortion with multiple complications and maternal death [4, 10]. In Africa 60% of unsafe abortions generally occurs in women below age 25 years and 40% occurs in the adolescent age group. Based on this evidence, evaluation of post-abortion care is required to ensure up-to-date health care of young women of reproductive age in African settings [11].

The burden of abortion in Botswana may be underestimated as the incidence of abortion is not well documented or known, due to the restrictive abortion law of the country. Botswana achieved a contraception prevalence rate of 53% which can be indirect evidence of high unmet need with its consequences of unintended pregnancy [12]. Women with unintended pregnancies are likely to have unsafe abortions leading to complications[13]. From the 2010 maternal death review in Botswana and the report of the Central Statistics Office of Botswana, maternal mortality due to abortion complication stands among the three leading causes of maternal death [14, 15].

There is a need to understand the degree and nature of post-abortion complication and its management in different settings. This study was aimed at generating baseline evidence on the presentations of post-abortion complications in selected hospitals in Botswana. The results will provide both clinicians and policy makers with information needed to improve post-abortion care, with the objective of decreasing the resultant maternal mortality.

## Methodology

### Study area

Botswana has a total population of 2.02 million inhabitants with female to male ratio of 1.047. Half of the female population is in the reproductive age group (15–49 years). According to the Botswana Central Statistics Office report of 2011 the total fertility rate in Botswana is 2.9 [16, 17].

Botswana has a total of 28 public hospitals; 3 referral (one psychiatric and two general), 10 district and 15 primary hospitals. According to the Central Statistics Office report of 2012 more than 55% of all deliveries were attended in district and referral hospitals [15]. This report also states that abortion complications accounted for 60–65% of admissions to Gynaecology wards. The data were collected from four hospitals in different districts–two referral hospitals, Princess Marina Hospital (PMH) and Nyangabgwe Hospital; and two district hospitals, Letsholathebe II Memorial Hospital in Maun district and Mahalapye District Hospital.

### Study design

A retrospective institution based cross-sectional study was conducted at selected hospitals. Samples were allocated to the different hospitals using a proportional sampling technique. All records of threatened abortion, ectopic pregnancy, molar pregnancy and other abnormal uterine bleeding were excluded from the study.

### Sampling and sample size

The hospitals for the study were selected based on convenience, representing both referral and district hospitals. Epi-Info 3.5.3 software was used to calculate the sample size. With the assumptions of prevalence of moderate to severe abortion complications of 28.1% [18], 5% margin of error and 5% level of significance, a sample size of 310 was obtained. To address the clustering effect in the different hospitals due to the difference in the characteristics of catchment populations as well as the peculiarities of quality of abortion care in the respective hospitals, the calculated sample size was multiplied by two (design effect). Hence the sample size was 620. Systematic random sampling was employed to select the charts to be reviewed from each hospital. The hospital register was used as a sampling frame.

Data were extracted from patients’ records with regards to socio-demographic variables, and abortion complications such as haemorrhagic shock and end organ damage, severe anaemia, septic shock, septic abortion, uterine perforation, peritonitis, pelvic abscess, hospital stay. The following information on patient management and outcomes were collected: diagnosis, intervention interval, standard of care, use of blood products, operative interventions, means of uterine evacuation, administration of antibiotics, counselling and post abortion family planning, and abortion related maternal death.

### Data collection

A structured data extraction sheet was used to collect data from patients’ records. The data collection sheet was pretested and standardization was done. The data collectors were trained. Data were collected by clinicians and midwives who are part of the team of investigators; completeness and accuracy of the data was checked. The pattern of complications was categorized according to South African researchers’ classification of abortion complications as follows:

**Low severity** (all criteria to be present): temperature <37.2°C, no suspicious finding on evacuation, no sign of infection and no system or organ failure.
**Moderate severity:** temperature 37.3–37.9°C or offensive conceptus tissue or localized pelvic peritonitis.
**High severity:** temperature > 38°C, organ or system failure, generalized peritonitis, septic shock, pulse>120 beats/minute, foreign body or sign of mechanical injury on evacuation [19].

### Data management and analysis

SPSS 22 software was used for data entry, cleaning and analysis. Frequency, percentage, means, standard deviation, and odds ratios with 95% confidence interval were computed to present the findings. Bivariate analysis was done to assess the association of the different independent variables with abortion complications. A p-value less than 0.05 was considered statistically significant.

### Ethical issues

Ethical clearance was secured from the institutional review board of the University of Botswana, the Ministry of Health and ethical committees of the study hospitals. Patients had already been managed as per the management protocol and they were not in the hospital at the time of data extraction. Hence, an informed consent waiver was granted. Patient identifying information was not included in the data extracted from the records.

## Results

Data were extracted from 620 files of which one file was excluded from the analysis because of incompleteness. Data from the remaining 619 files were included in the analysis making 99.8% coverage of the calculated sample size. The mean (SD) age was 27.12 (5.97) years with range of 13–46 years. Of the 619 study population, 8.2% were teenagers while 36.8% were aged less than 25 years. Regarding marital status, 81.3% were single while 10% being married (Table 1).

**Table 1:** Background and reproductive characteristics of the patients admitted to hospitals.

Characteristics	Frequency	Percentage
**Age in Years**		
13–19	51	8.2
20–24	177	28.6
25–29	185	29.9
30–34	130	21
35 & above	76	12.3
Age range: 13–46	Mean (SD): 27.12 (5.97)	
**Marital status**		
Single	503	81.3
Married	62	10.0
Divorced	4	0.6
Widowed	1	0.2
Separated	1	0.2
Unknown	48	7.7
**Parity**		
Zero	58	9.4
One	161	26.0
Two	180	29.1
Three	129	20.8
4 & above	91	14.7
**Number of abortions**		
One	460	74.3
Two	151	24.4
3 & above	8	1.3
**Current pregnancy status**		
Planned	36	5.8
Unplanned but wanted	16	2.6
Unplanned and unwanted	25	4
Undocumented	542	87.6
**Causes of abortion**		
Spontaneous	591	95.5
Induced by the patient	24	3.9
Induced for medical indication	4	0.6
**Gestational age by LNMP**		
Up to 12 weeks	324	52.3
13–24 weeks	203	32.8
Unknown	92	14.9

Three quarters of the patients were having their first pregnancy loss while 159 (25.7%) had two or more pregnancy losses. Of the 159 cases with recurrence, 38 (24%) of the consecutive losses had occurred within six months of the previous pregnancy loss. The large majority of abortion cases (95.5%) were reported to be spontaneous while 3.9% of the abortions were induced by the patient. More than half (52.3%) of the patients were admitted as a case of first trimester pregnancy loss. Unplanned pregnancy was reported in 6.6% of cases (Table 1).

Two thirds of the patients were admitted at their first visit to the hospitals and one third were referrals from other health facilities. The majority (66.4%) of the patients were admitted as cases of incomplete abortion followed by inevitable abortion (16.8%). Offensive discharge (17.9%), tender uterus (11.3%), septic shock (3.9%) and pelvic peritonitis (2.4%) were among the physical findings recorded at admission (Table 2).

**Table 2:** Admission characteristics of the patients admitted to hospitals.

Admission characteristics	Frequency	Percentage
**Status of admission to the hospital**		
Direct first visit to the hospital	344	55.6
Readmission to the same hospital	30	4.8
Referral from other institute	211	34.1
Self-referral after prior visits at other health facilities	3	0.5
Not documented	31	5.0
**Admission type of abortion**		
Inevitable	104	16.8
Incomplete	411	66.4
Complete	23	3.7
Missed abortion	40	6.5
Blighted ovum	17	2.7
Not documented	24	3.9
**Sign of infection**		
Offensive discharge	111	17.9
Tender uterus	70	11.3
Septic shock	24	3.9
Pelvic peritonitis	15	2.4
Pelvic abscess	4	0.6
Uterine perforation	4	0.6
Bowel perforation	4	0.6
Generalized peritonitis	7	1.1
**Other findings**		
Tablet in the vagina	3	0.5
Foreign body in the vagina	11	1.8
Bruise/laceration of the Cervix/vagina	7	1.1

**Fig 1:**
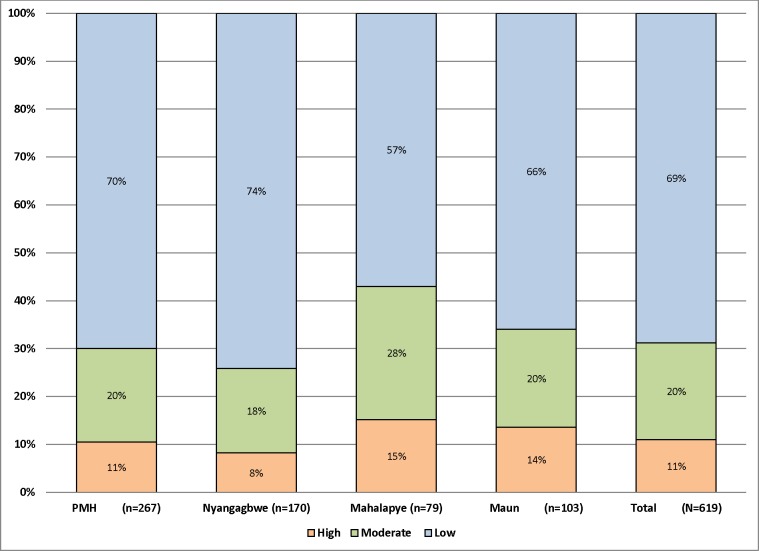
The severity of patients presenting with abortion at the hospitals.

The patients were classified according to the level of severity of abortion using South African researchers’ classification as described above. It was found that 68 (11%) of them were of high, 125 (20.2%) of moderate and 426 (68.8%) of low severity (Fig 1).

More than a third (36.5%) of the patients presented to health facilities three days after onset of vaginal bleeding. In 27% of cases the planned uterine evacuation was done after 24 hours.

Anaemia confirmed with haemoglobin (HB) values less than 11g/dl was the leading complication. Of 252 patients whose HB was recorded, 54.8% had HB levels of less than 11g/dl and clinically significant anaemia with HB<7g/dl in 14.9%. Clinically 193 out of 619 (31.2%) patients were pale. Clinical detection of pallor was a good predictor of laboratory confirmed anaemia (HB<11g/dl) in 79.3% of the cases, whereas 32.1% of the cases who were not labelled pale clinically had HB<11g/dl (p<0.001). Anaemia (HB<11g/dl) in HIV positive and HIV negative patients was 56.6% and 47% respectively; the difference was not statistically significant (p = 0.22). Hypovolemic and septic shock was noted in 65 (10.5%) of the cases.

Other complications included multiple organ failure (1.6% of cases), disseminated intravascular coagulation (DIC) in eight cases (1.3%), renal failure in five (0.8%), ARDS in three (0.5%) and hepatic failure two (0.3%) patients. There were a total of nine abortion related deaths with a case fatality rate of 1.5%: one patient with unsafe induced abortion, two uterine perforations, one pelvic abscess, two generalized peritonitis and seven septic shocks. Even though seven out of nine deaths were from second trimester and the other two were from first trimester, there was no significant statistical association between the death and gestational age (p = 0.11).

All of the patients received either prophylactic or therapeutic antibiotics, where more than half 346(55.9%) were covered with therapeutic course. Surgical or medical means of uterine evacuation was employed in 550 (88.9%) patients. Oxytocin and misoprostol was administered to 86 (13.9%) and 50 (8.1%) of cases concurrently with surgical evacuation. Metallic curette was used in 516 (83.4%) patients and vacuum aspiration in 18 (2.9%) cases. In 140 (27%) of cases, uterine evacuation was done 24 hours after admission. Hysterectomy, laparotomy, and bowel surgery was done to seven (1.1%), six (1%) and three (0.5%) of the cases respectively. Blood products were administered in 59/619 (9.5%) of the patients. Post abortion counseling was provided to 121 (19.5%) while 30(4.8%) patients received some form of contraceptive method. Hospital stay beyond 24 hours was noted in more than one third 198(36.7%) of cases for inpatient care of whom ten (1.6%) were admitted to ICU.

Bivariate analysis was done to examine an association between independent variables and abortion related complications. Induced abortion was significantly associated with abortion complications (p = 0.018). Patients admitted in one of the district hospitals (Mahalapye) and a referral hospital (PMH) tended to develop abortion complications more than the patients in the other two hospitals: OR 95%, CI = 2.72 (1.45, 5.09) and 2.20 (1.34, 3.62) respectively. Delay in the evacuation of the uterine contents for more than six hours was associated with increased abortion related complications (p = 0.035). In patients with a parity of four and above marginal association with post-abortion complications was found (p = 0.053) (Table 3).

**Table 3:** Bivariate analysis of factors associated with abortion complications.

Variables	Abortion complications N (Row %)		Crude Odds ratio (95% CI)	P-value
**Age in Years**	**Yes**	**No**		
13–20 years	15 (17.2)	72 (82.8)	Reference	
Over 20 years	134 (25.2)	398 (74.8)	1.62 (0.89,2.92)	0.111
**Marital status**				
Single	113 (22.5)	390 (77.5)	Reference	
Married	12 (19.4)	50 (80.6)	0.83 (0.45,1.61)	0.578
**HIV status**				
Negative	69 (21.9)	246 (78.1)	Reference	
Positive	41 (28.9)	101 (71.1)	1.45 (0.92, 2.27)	0.108
**Duration of vaginal bleeding**				
One day	56 (21.6)	203 (78.4)	Reference	
2–3 days	21 (20.4)	82 (79.6)	0.93 (0.53, 1.63)	0.796
4 days and above	28 (28.6)	70 (71.4)	1.45 (0.86, 2.46)	0.168
**Gestational age**				
First trimester	70 (21.6)	254 (78.4)	Reference	
Second trimester	35 (17.2)	168 (82.8)	0.76 (0.48, 1.19)	0.223
**Number of previous abortions**				
Zero	98 (24.6)	300 (75.4)	Reference	
One	46 (25.4)	135 (74.6)	1.04 (0.69, 1.56)	0.838
2–3	5 (12.5)	35 (87.5)	0.44 (0.17, 1.15)	0.093
**Causes of abortion**				
Spontaneous	122 (21.6)	444 (78.4)	Reference	
Induced	8 (47.1)	9 (52.9)	3.24 (1.22, 8.56)	0.018
**Classification of abortion**				
Inevitable	26 (25.0)	78 (75.0)	Reference	
Incomplete	88 (21.4)	323 (78.6)	0.82 (0.49, 1.35)	0.431
Complete	6 (26.1)	17 (73.9)	1.06 (0.38, 2.97)	0.913
Missed	12 (30.0)	28 (70.0)	1.29 (0.57, 2.89)	0.543
**Hospital**				
Nyangabgwe	26 (15.3)	144 (84.7)	Reference	
Mahalapye	26 (32.9)	53 (67.1)	2.72 (1.45, 5.09)	0.002
Letsholathebe II Memorial	21 (20.4)	82 (79.6)	1.42 (0.75, 2.68)	0.281
PMH	76 (28.5)	191 (71.5)	2.20 (1.34, 3.62)	0.002
**Hospital stay before evacuation**				
0–6 hours	39 (18.9)	167 (81.1)	Reference	
Over 6 hours	110 (26.6)	303 (73.4)	1.56 (1.03, 2.35)	0.035
**State of hospital visit**				
First visit	75 (21.8)	269 (78.2)	Reference	
Repeat	7 (23.3)	23 (76.7)	1.09 (0.45, 2.64)	0.846
Referral from other facility	48 (22.7)	163 (77.3)	1.06 (0.70, 1.59)	0.794

## Discussion

The study demonstrated that a significant proportion of the cases of abortion had anaemia and signs of infection which were linked to bleeding and possibly induced abortion. The high rate of anaemia (HB<11g/dl) in this study (52.4%) is consistent with WHO estimates of anaemia in pregnant women of Botswana, given as 13–57%. Clinically detectable anaemia with pallor was observed in 31.2% of patients and significant anaemia with HB<7g/dl (which is an indication for transfusion in our setting) in 14.9%. Such high rates of anaemia was not related to HIV infection (p = 0.22) and could rather be ascribed to prolonged bleeding coupled with delayed patient presentation [20]. Of the total patients in this study, only 9.5% received transfusion [21]. The severe forms of abortion complications were significantly higher among patients who presented with induced abortion and in patients with delayed uterine evacuation. There was a significant difference between hospitals in the incidence of abortion related complications, which might be a result of delayed referral processes and initiation of appropriate clinical care. Almost all patients reported spontaneous abortion, which did not tally with the severity of patients’ condition and the abortion complications witnessed in the hospitals. It is likely that patients with induced abortion might have claimed that it is spontaneous due to its legal implications or social desirability bias.

The majority (95.5%) of the patients were admitted as cases of spontaneous abortion and only 3.9% of the patients reported to have induced pregnancy loss by themselves, either by insertion of a foreign body into the vagina or taking unspecified tablets. In 21 (3.4%) cases evidence of interference in the cervix and/or vagina had been detected and only six of these patients reported to have spontaneous abortion. Literature has shown that spontaneous miscarriage is higher in women with unintended pregnancy because the women are usually engaged in high risk behaviours to facilitate pregnancy loss [22]. Even then this finding should be interpreted carefully as it underestimates self-induced abortion. In a country with restrictive abortion laws the care provider may not document the information in the patient`s medical record or the patient may not report the interference for fear of prosecution. In 2006 a similar finding of 4% self-induced abortion was reported in Syria, another country with restrictive abortion laws [3]. The study findings reveal lower figures than a study conducted in Ethiopia after legalization of abortion in that country, which reported 14% self-induced abortion and 7% mechanical injury as a sign of interference [23]. One third of patients in Kenya also reported that they had interfered with their pregnancy [24].Two other studies in Ethiopia and Tanzania have reported that out of all the patients admitted as spontaneous abortion, 53% and 60% of those who responded to confidential interview had induced the pregnancy loss. The Tanzanian study also reported that 60% of all self-induced abortion was done after 12 weeks of gestation [25, 26]. The high incidence of second trimester abortion which is 32.8% in our study is inconsistent with spontaneous miscarriage since 80% of spontaneous pregnancy loss is expected to occur in the first trimester [27]. Our finding is consistent with the 38–40% incidence of second trimester abortion in a study done in Ethiopia after legalization of abortion. The finding of such high incidence of second trimester loss may be due to intervention by the patient [23, 28]. In countries with restrictive abortion laws women with induced abortion will present as a case of spontaneous incomplete abortion for fear of stigma and accountability unless there is confidential interview [25, 26].

One in five patients presented with offensive vaginal discharge as the commonest sign of infection followed by pelvic tenderness and septic shock. Other life-threatening complications included uterine perforation, bowel perforation with generalized peritonitis. All these serious complications are not a feature of simple spontaneous miscarriage.

Our patients were categorized into three levels according to severity of abortion complications as follows; low 68.8%, moderate 20.2% and high severity 11% (Fig 1). This finding is consistent with a study done in South Africa in 1994 found a low level of severity in 66.4% of cases, a moderate level in 18.6% and a high level in of 15% [19]. Another study done in the same country after legalization of abortion in 2003 reported low levels of complications at 72.3%, moderate levels at 18.4% and high severity at 9.7%–thereby revealing a moderate reduction in high severity complications[18]. A third study in South Africa in 2005 reported further reduction of morbidity without a change in the incidence of incomplete abortion [29].

Thirty-four per cent (34.1%) of patients were admitted as referrals from other health institutions. More than one third of patients sought medical care after three days of vaginal bleeding which is an uncommon practice for women with wanted pregnancy. For one third of the patients the planned uterine evacuation was done after 24 hours. Such late presentation by the patient coupled with a referral process and delayed initiation of a planned medical care might contribute to the higher complications and mortality rates. A study done by Ziraba et al. revealed that delayed presentation or care is associated with increased incidence of abortion related complications [30].

There were a total of nine deaths with a case fatality rate of 1.5% with significant evidence of unsafe induced abortion like use of foreign bodies, vaginal laceration and others. This is higher than the finding in a study in 2005 in Kenya (where abortion is also illegal) which reported a 0.87% fatality rate [31]. This can be explained by a methodological difference since the Kenyan study had included all levels of health facilities, whereas our study was conducted mainly in hospitals; patients presenting at such facilities are most likely to be in a more critical condition. An earlier study conducted in three other African countries where abortion is also illegal (Benin, Cameroon and Senegal) reported a case fatality rate of 2.3% in patients with induced abortion and 0.4% in those with spontaneous miscarriage [32]. The majority of cases (95.5%) were reported to be spontaneous abortion which again is not congruent with the high case fatality rate seen in the study, suggesting the likelihood of under-reporting of induced abortion due to fear of its legal consequences. Based on the United Nations Process Indicators of Emergency Obstetric Care the case fatality rate due to direct obstetric complications should not exceed 1%; however no standard value for acceptable case fatality rate specific to abortion complications could be found [33]. According to evidence from USA and Romania legalization of abortion has dramatically decreased maternal mortality due to post-abortion complications. Historically Romania banned legal abortion in 1966 with the intention of increasing the birth rate, but this measure resulted in increased maternal mortality without necessarily improving the birth rate. The impact of legalization of abortion and use of contraception in 1989 in this same country decreased abortion related maternal mortality by 50% over one year period [34–36]. This is also the case with Botswana's neighbour South Africa, where the annual number of abortion-related deaths fell by 91% after the liberalization of the abortion law in 1996 [36].

In the present study self-induced abortion by the patient was shown to be statistically associated with post-abortion complications. This is consistent with findings from Ethiopia and Kenya which revealed that interference with pregnancy was a significant predictors of fatal outcome [24, 28]. Over two fifths of the patients at Mahalapye were in the abortion complications category of moderate to high severity at the time of presentation. Such a high rate of complication in Mahalapye (one of the district hospitals) warrants further investigation that might provide insight into quality of post-abortion care in other similar level hospitals in the country. The high severity at presentation in Mahalapye could be a reflection of higher level of abortion complications in the community. Studies in Kenya and South Africa have shown differences between regions and different levels of hospitals in terms of use of low cost technology in the management of post-abortion complications. In the South African setting, training health care providers in the use of low cost and effective technology like manual vacuum aspiration and misoprostol has replaced metallic curettage and general anaesthesia. This simple technology shortens the time to uterine evacuation and addresses the delay in care which was significantly associated with post-abortion complication in this study [18, 24]. The higher complication rate in PMH could be a result of delayed initiation of patient care due to the referral process, since referrals constitute more than one third of admissions; a further possible cause is receiving complicated cases from other health facilities. It is likely that patients with such presentations might have induced abortion and sought clinical care only after their condition got worse. The marginal association of complications with a parity of four or more may be a result of unsafe induced abortion to limit the number of children which might be consistent with the fertility rate of 2.9 in Botswana. The fact that abortion is not legalized might influence people to seek clandestine abortion or self-induction with resultant complications. The result showed that two fifths of the patients were either referred from other facilities or were re-admitted at the hospitals, which might highlight an element of delayed care.

The study has the following limitations. Being a retrospective study, incompleteness of information was a challenge. The psychological impact of abortion/miscarriage was not assessed. It was difficult to know whether the pregnancies were planned or not in the majority of cases. As our study did not include a confidential patient interview, it is likely that there was an under-estimation of the level of induced abortion.

## Conclusion

Abortion related complications and deaths are high in our setting where abortion is illegal. The high incidence of second trimester miscarriage and repeat pregnancy loss are likely to be related to unsafe induced abortion. Unsafe induced abortion and delayed initiation of uterine evacuation had a significant impact on the incidence of severe post-abortion complications. A mechanism needs to be devised in the health facilities for timely evacuation of the uterus whenever it is indicated using simple technology like vacuum aspiration instead of metallic curette, and to be equipped to handle potentially fatal complications. Referrals constitute one third of admissions which may delay patient presentation and contribute to the high complication rate. Decentralization of post-abortion care services and regular clinical audit are recommended to avoid delayed initiation of patient care. This finding might help policy makers to consider revision of the abortion law. Standardizing the patient management protocol that is designed for all levels of complications and facilities, and its subsequent implementation, can reasonably be expected to decrease the impact of abortion related complications. A prospective study with a confidential interview on abortion will provide a better understanding on the incidence of induced abortion, its causes and complications.
